# Dispersion engineering with plasmonic nano structures for enhanced surface plasmon resonance sensing

**DOI:** 10.1038/s41598-018-27023-x

**Published:** 2018-06-13

**Authors:** Pankaj Arora, Eliran Talker, Noa Mazurski, Uriel Levy

**Affiliations:** Department of Applied Physics, The Benin School of Engineering and Computer Science, The Center for Nanoscience and Nanotechnology, The Hebrew University of Jerusalem, Jerusalem, 91904 Israel

## Abstract

We demonstrate numerically and experimentally the enhancement of Surface Plasmon Resonance (SPR) sensing via dispersion engineering of the plasmonic response using plasmonic nanograting. Following their design and optimization, the plasmonic nanograting structures are fabricated using e-beam lithography and lift-off process and integrated into conventional prism based Kretschmann configuration. The presence of absorptive nanograting near the metal film, provides strong field enhancement with localization and allows to control the dispersion relation which was originally dictated by a conventional SPR structure. This contributes to the enhancement in Q factor which is found to be 3–4 times higher as compared to the conventional Kretschmann configuration. The influence of the incident angle on resonance wavelength is also demonstrated both numerically and experimentally, where, only a negligible wavelength shift is observed with increasing the incident angles for plasmonic nanograting configuration. This surprising feature may be helpful for studying and utilizing light-matter interaction between plasmons and narrow linewidth media (e.g. Rb atom or molecule) having nonlocalities in their susceptibility-momentum relation. Finally, we analyze the role of plasmonic nanograting in enhancing the performance of an SPR sensor. Our results indicate that the integrated SPR-nanograting device shows a great promise as a sensor for various types of analytes.

## Introduction

Surface Plasmon Polariton (SPP) is an electromagnetic wave propagating along a metal-dielectric interface, coupled to oscillations of electrons in the metal. The coupling of light into SPPs requires phase matching, where the phase velocity of the surface plasmon wave and of the lateral component of the incident light become equal^1,2^. Being localized at the interface, the SPP wave is extremely sensitive to minute changes in refractive index of the dielectric medium, in the vicinity of the interface^3,4^. One of the most common techniques to excite the SPP for sensing is the Kretschmann configuration^5,6^, where the signature of SPP excitation results in a decrease in the intensity of the reflected light from the metal surface for a particular resonance angle or wavelength^7–9^. Commonly, to achieve high sensitivity, the resonance linewidth should be as narrow as possible. Unfortunately, plasmonic resonances are typically broad, mostly due to Ohmic loss in the metal^10^. In this regard, coating the Kretschmann configuration prism by thin multilayers was recently reported theoretically and shown experimentally to allow the engineering of the dispersion curve^11,12^. Periodic structures have been shown to provide better figure of merit for sensing via dispersion engineering and in particular via the increase in group index, leading to the narrowing of the resonance linewidth^13,14^. Indeed, enhanced sensitivity detection based on dispersion engineering using metallic gratings applied upon thin metal coated prism has been reported theoretically^15–18^, but to this end, no experimental results have been reported demonstrating the enhanced sensitivity with the metal grating on conventional prism configuration. Hereby, we demonstrate both numerically and experimentally the use of plasmonic nanogratings to engineer the dispersion characteristics of SPPs with the goal of obtaining narrow resonance linewidth and thus enhancing sensing capabilities. Moreover, as a result of the dispersion engineering, we observe a negligible wavelength shift with changing the incident angle. As a result, the proposed plasmonic nanograting device can be used as a robust system to keep the resonance wavelength constant for broad angular (momentum) range without shifting away of the resonance frequency of the media. Certainly, the proposed structures can also be used for grating coupled plasmon based sensor devices, where the light is directly illuminated on the gratings to couple a desired diffraction order to SPP mode instead of the prism coupling^19,20^. However, grating coupled plasmon based sensors have been shown to exhibit lower sensitivities compared to prism-coupled based sensors due to distribution of incident light across multiple diffraction orders^21,22^. The major drawback of grating coupled plasmon based sensor in sensing applications is that, the light is incident through the sample solution and therefore the analyte and flow cell needs to be optically transparent. On the other hand, while the interrogation optical system for both the SPR sensors are essentially the same, the accurate control of thin plasmonic metal layer is not required in grating coupled based sensor as compared to prism coupled based sensor.

**Figure 1:**
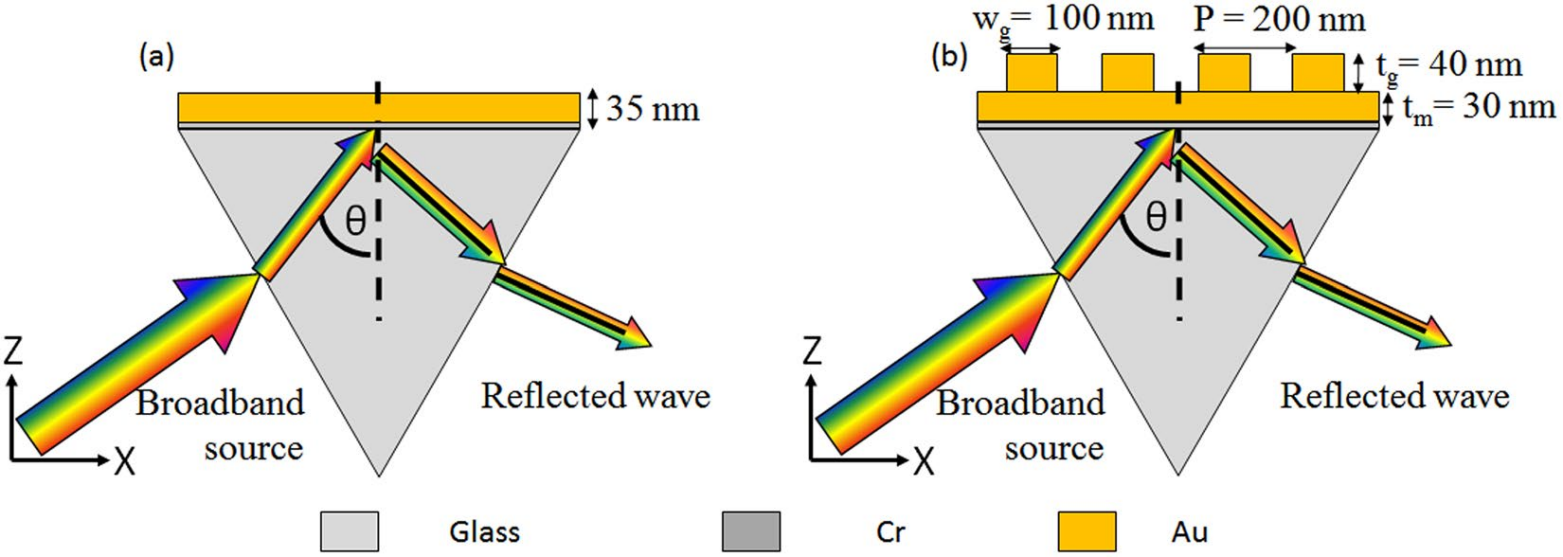
Schematic of (**a**) Conventional and (**b**) Plasmonic nanograting based Kretschmann configuration. A 2 nm Cr adhesion layer followed by a 35 nm thick gold film is deposited on the prism. Rectangular gold nanogratings with a thickness (t_g_) of 40 nm, a period (P) of 200 nm and a width (w_g_) of 100 nm are fabricated on top of 30 nm thick gold film.

Throughout the paper, we are comparing the conventional Kretschmann configuration (Fig. 1a), with our configuration which is based on incorporating a nanograting on top of the metal prism (Fig. 1b). SPR sensors are typically based on either monitoring the spectral (wavelength) response, or the angular response. In some cases, both spectral and angular response are combined. The combined interrogation approach effectively increases the spectral range simply by changing the angle of incidence using the same broadband source. As such, it allows for multispectral characterization of the sample^23,24^. Here, we are mostly focused on the spectral interrogation approach^25,26^, which is becoming more attractive due to the availability of cheap and miniaturized spectrometers and due to the importance in integrating the SPR sensor with absorption spectroscopy^27–30^. By integrating the plasmonic nanograting on top of the prism, we experimentally observed about 3-fold enhancement in Q factor compared to the conventional Kretschmann configuration. As a result, our configuration provides enhanced SPR sensing compared to conventional prism configuration. It should also be mentioned that other SPR sensors exist, e.g. based on Surface Enhanced Raman Spectroscopy (SERS) and single molecule Fluorescence Resonance Energy Transfer (smFRET). Yet, these sensors are outside the scope of the current paper.

## Results and Discussions

### Numerical simulations results

In the simulations, a CaF_2_ glass was used as the prism substrate (n = 1.4307) for both configurations. The wavelength dependent dielectric constant for gold was taken from previous publications^31,32^. A 2 nm layer of Cr (n = 3.9592–4.1936i) was also considered below the gold layer as an adhesion layer in the simulation to match the realistic experiment scenario. The thickness of thin metal layer for conventional Kretschmann configuration, shown in Fig. 1(a), was optimized using transfer matrix method^33^. The geometrical parameters for the configuration incorporating plasmonic nanograting on top of thin metal layer, which shown in Fig. 1(b), were optimized using RCWA method^34^. Broadband simulations were performed to extract the TM polarization based reflection characteristics and field distributions were obtained using a commercial finite elements full wave solver (Comsol Multiphysics).

**Figure 2:**
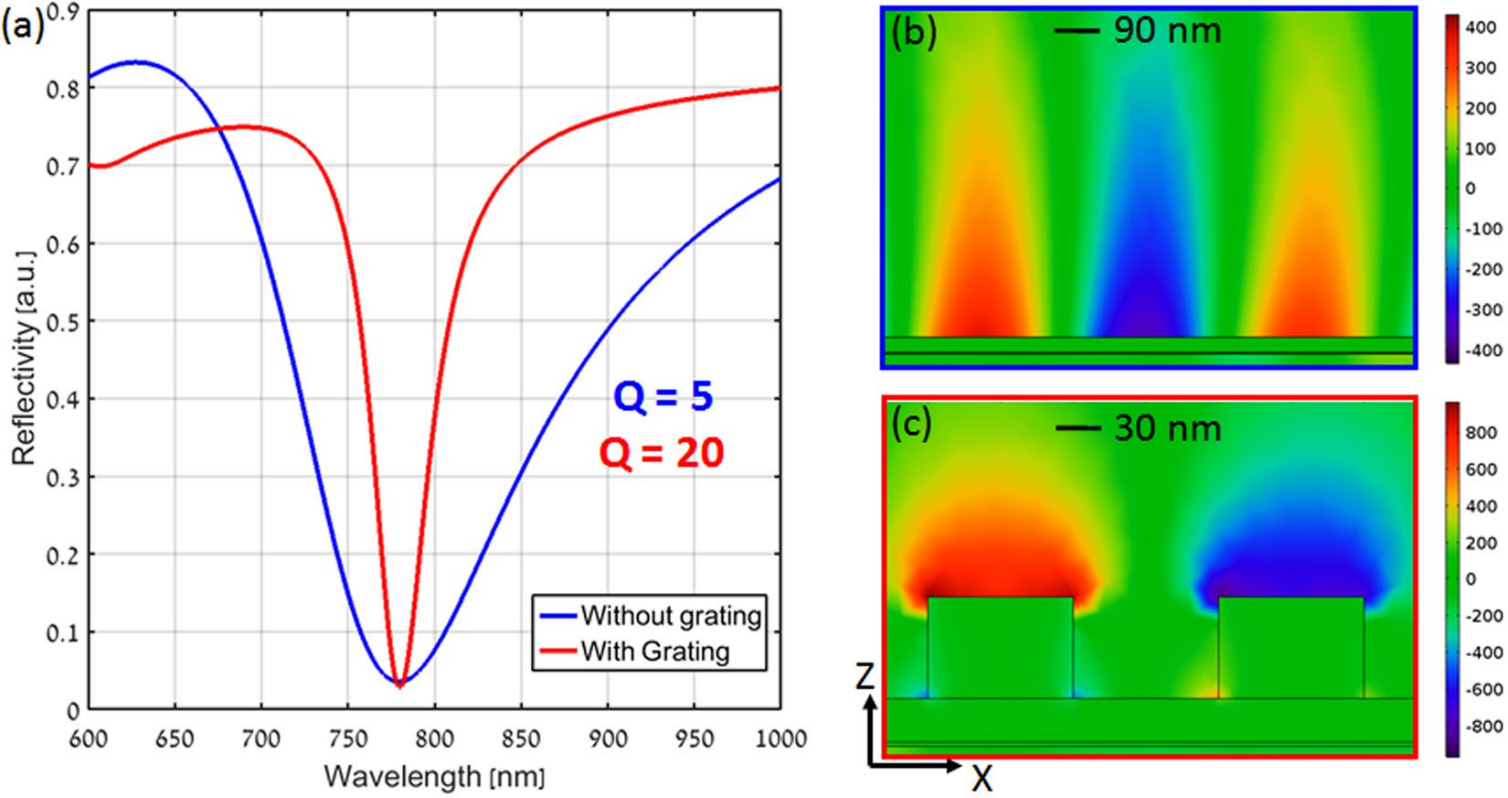
(**a**) Calculated reflectivity as a function of wavelength at a fixed incidence angle for conventional and plasmonic nanograting based Kretschmann configuration respectively. Electric field distribution for (**b**) conventional and (**c**) plasmonic nanograting based Kretschmann configuration. The different zoom in scale has been used for both the field plots for visualization purpose. Scale bar is arbitrary but similar normalization was applied for both panels b and c.

Figure 2(a) shows the TM polarization based reflection spectrum for a conventional prism configuration (blue line) with gold thickness of 35 nm, assuming a fixed incident angle of 45.97°. As shown, the dip in reflectivity at the wavelength of 780 nm is clearly observed. The Q factor was found to be ~5. The reflection spectrum obtained for an optimized plasmonic nanograting, on top of 30 nm thin metal layer, with thickness (t_g_) 40 nm, period (P) 200 nm and width (w_g_) 100 nm is also shown (red line) in Fig. 2(a). The angle of incidence was assumed to be 82°. As can be seen, a sharp dip at the same wavelength (780 nm) is observed in the reflectivity spectrum. The Q factor for the plasmonic nanograting device was found to be ~20, i.e. 4 times larger as compared to the conventional Kretschmann configuration. Figure 2(b,c) show the electric field distributions at the resonance wavelength (780 nm) for the conventional and the nanograting configurations respectively. In the case of plasmonic nanograting shown in Fig. 2(c), the field enhancement is higher as compared to the traditional prism configuration shown in Fig. 2(b). The existence of absorptive nanograting near the metal film leads to a perturbation in the dispersion relation of the conventional SPR structure and contributes to the enhanced Q factor^18,20^.

**Figure 3:**
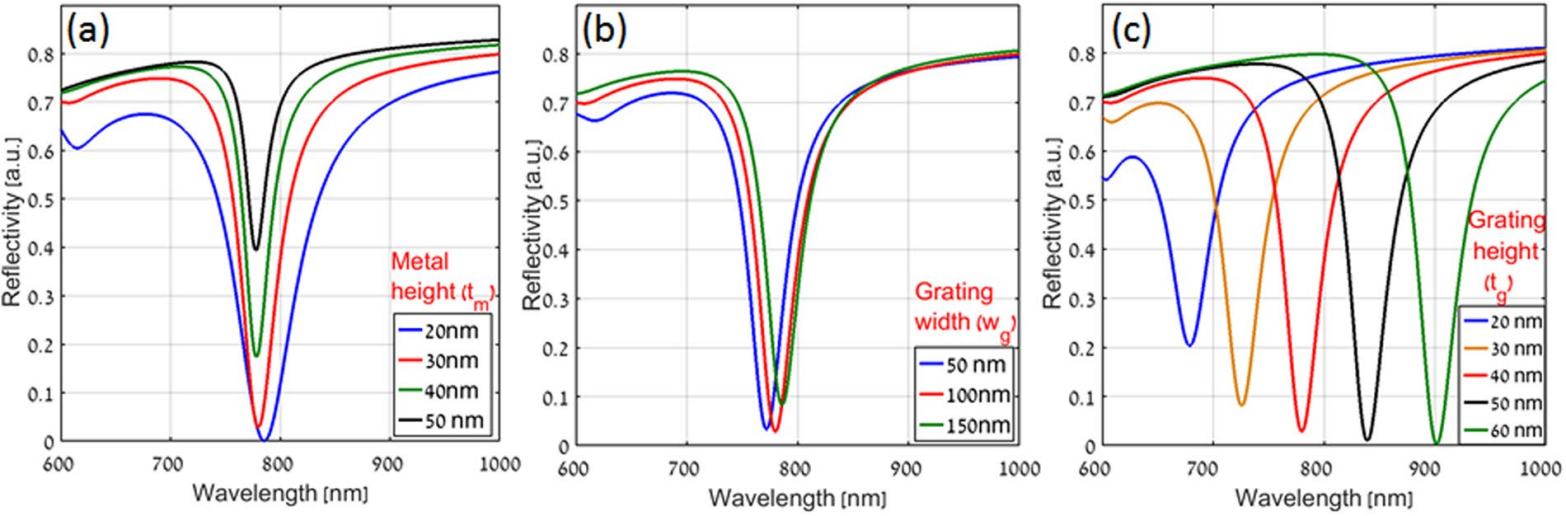
Calculated reflectivity as a function of wavelength for plasmonic nanograting based Kretschmann configuration at an incident angle of 82° for a grating period of 200 nm with (**a**) variation in thin metal height (t_m_) (**b**) variation in grating width (w_g_) and (**c**) variation in grating height (t_g_) respectively.

To get the optimized geometry for proposed plasmonic nanograting structure, the geometrical parameters namely the thin metal height (t_m_), grating width (w_g_) and grating height (t_g_) (labeled in Fig. 1(b)) were changed systematically and the reflectivity was measured for TM polarization for a range of wavelengths from 600 nm to 1000 nm at fixed incident angle of 82°as shown in Fig. 3. The final geometrical parameters were chosen during the simulations to achieve narrowest linewidth and high signal contrast.

Figure 3(a) shows the reflectivity spectra for t_g_ of 40 nm, P of 200 nm, w_g_ of 100 nm and with different thickness of metal height (t_m_). As t_m_ was increased towards 50 nm, the minimum reflectivity reduced up to 0.4 (black color) resulting in low signal contrast but with narrowest Full Width at Half maximum (FWHM). When t_m_ was lower than 30 nm (blue color), the reflectivity value was found to be the lowest but with higher FWHM. Hence, a careful choice of the thickness of the thin metal (t_m_) of 30 nm (red color), resulted in a decent value of signal contrast and FWHM.

Figure 3(b) shows the reflectivity spectra for t_m_ of 30 nm, P of 200 nm, t_g_ of 40 nm and with different width of metal grating (w_g_). It was noticed that the value of 100 nm (red color) for grating width resulted in a high signal contrast and narrow FWHM.

Figure 3(c) shows the reflectivity spectra for t_m_ of 30 nm, P of 200 nm, w_g_ of 100 nm and with different thickness of metal grating height (t_g_). It was noticed that the SP resonance wavelength can be easily tuned by changing the grating height and the increase in the grating height leads to high signal contrast together with a lower FWHM in the reflectivity spectra. So, the metal grating height was optimized to t_g_ = 40 nm (shown in red color) for SP resonance wavelength of 780 nm.

So, from Fig. (3), It was observed that metal height (t_m_) of 30 nm, grating width (w_g_) of 100 nm and grating height (t_g_) of 40 nm, exhibits an optimum compromise between high signal contrast and narrow FWHM for SP resonance wavelength of 780 nm.

**Figure 4:**
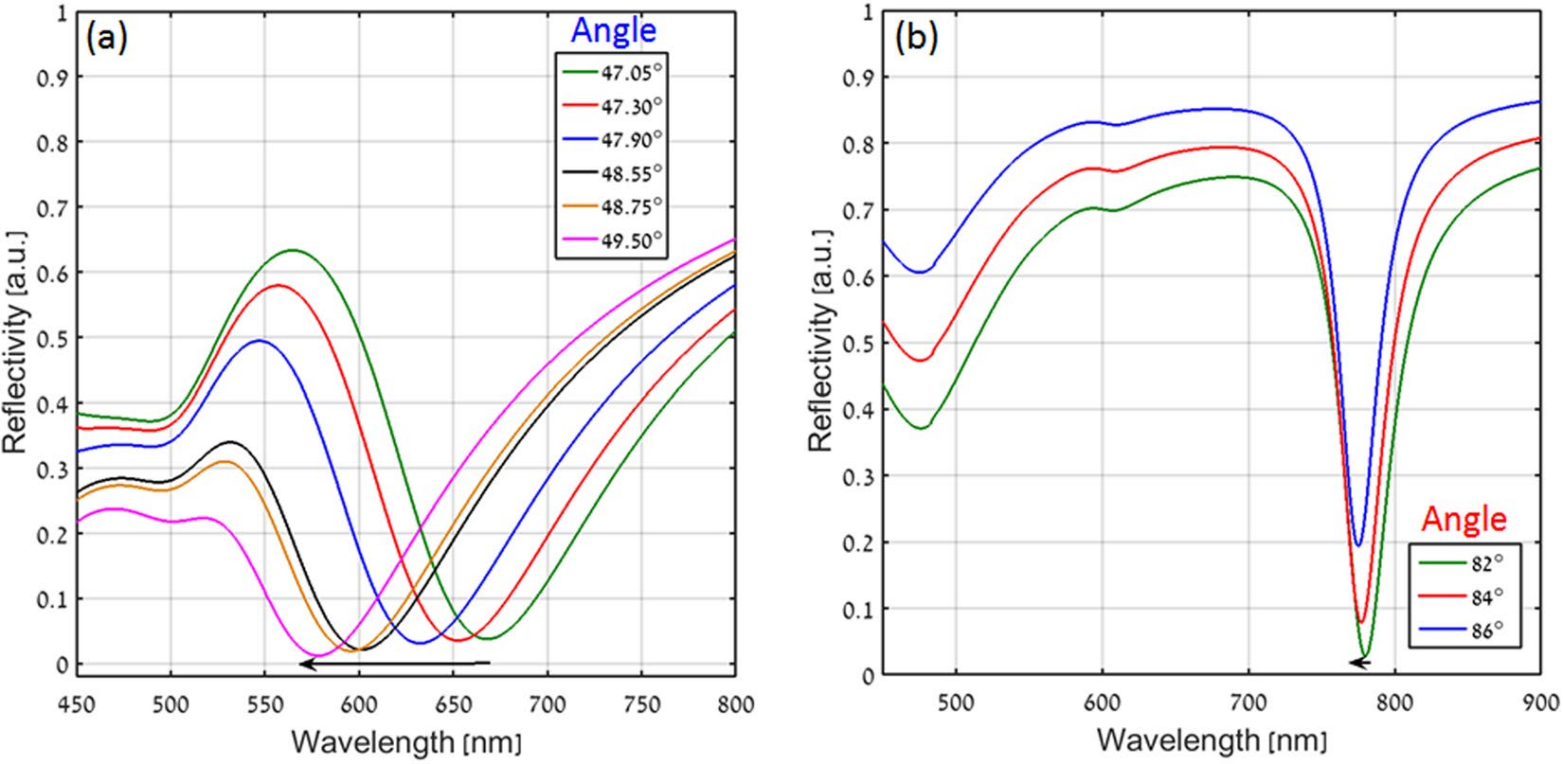
Calculated reflectivity as a function of wavelength with variation in incidence angle for (**a**) Conventional and (**b**) Plasmonic nanograting based Kretschmann configuration.

In order to find out the relation between the angle of incidence and the resonance wavelength, the reflectivity spectra of both the configurations were calculated for different incident angles. While the relation for the conventional Kretschmann configuration is well known and can be easily derived from simple phase matching condition, it turns out that this is not the case for our nanograting based device. This is due to the strong dispersion imprinted by the plasmonic nanograting. Figure 4(a,b) show the reflected spectra for various angles of incidence in both the configurations respectively. As can be seen, in the case of Fig. 4(a) the resonance wavelength decreases significantly with the increase in incident angle, following the relation:

1 $${K}_{in}||=\frac{2\pi }{\lambda }{n}_{prism}\,\sin \,\theta ={K}_{spp}=\frac{2\pi }{\lambda }{n}_{spp}$$

where ${K}_{in}||$ is the momentum of the incident wave in the parallel (x) direction, *θ* is the angle of incidence, *n*_*prism*_ is the refractive index of the prism, *λ* is the resonance wavelength and *n*_*spp*_ is the refractive index of the plasmonic mode. Yet, while the resonance wavelength for the case of the plasmonic nanograting based configuration must also follow the same rule, one observes a negligible wavelength shift for different incident angles, as shown in Fig. 4(b). This striking observation is the direct result of our dispersion engineering approach, implemented by the plasmonic nanograting. This behavior can be understood by taking the derivative of Eq. 1 with respect to the wavelength. By doing so, one obtains

2 $$\frac{d\theta }{d\lambda }=\frac{1}{{n}_{prism}\,\cos \,\theta }\frac{d{n}_{spp}}{d\lambda }$$

As can be seen, it is possible to minimize the angular sensitivity of the resonance wavelength (i.e. to maximize *dθ*/*dλ*), by increasing the dispersion *dn*_*spp*_/*dλ*)^35^. Indeed, we find a striking difference in group index [(defined as ${n}_{g}={n}_{spp}-\lambda \frac{d{n}_{spp}}{d\lambda }$) between the two configurations, where the nanograting approach yields a group index of 4.18, whereas the group index of the conventional Kretschmann approach is only 1.17.

**Figure 5:**
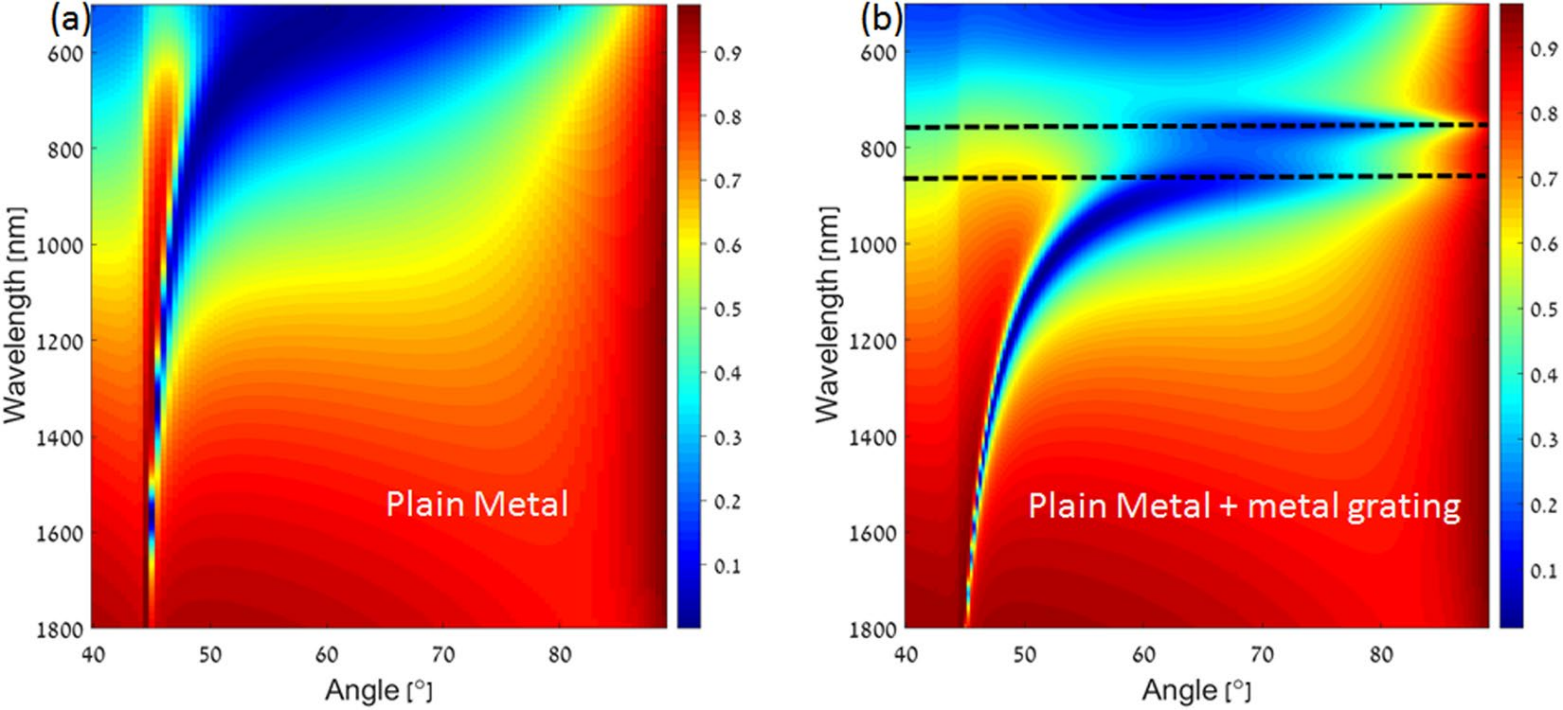
2D dispersion curve for (**a**) Conventional and (**b**) Plasmonic nanograting based Kretschmann configuration. The color bar indicates the reflectance values. Dashed lines are added to show the dispersive plasmonic modes. The upper dashed line is originated from the nanograting while the lower dashed line is originated from the conventional Kretschmann configuration. As can be seen, the addition of the nanograting adds significant dispersion to the system, allowing to obtain high group index and narrow resonances.

It has been already shown that effective dispersion engineering in the composite structures can be achieved by patterning the metal layer^36–38^. To learn more about the dispersion characteristics for both the configurations, we have calculated the reflectivity of both configurations as a function of wavelength and incident angle. Wavelength scanning from 400 nm to 1800 nm with an increment of 0.05 nm and angle scanning from 40° to 89° with an increment of 0.25° are performed. The obtained results are shown in Fig. 5(a) (Kretschmann configuration) and 5(b) (nanograting configuration). The deep blue regions indicate minimum in reflectivity, corresponding to the excitation of surface plasmon resonance. For Fig. 5(a), one can observe the standard dispersion curve for conventional Kretschmann configuration. In striking contrast, the introduction of a nanograting on top of the flat metal brings about a drastic change in reflection characteristics as shown in Fig. 5(b). In general, the resonances in this dispersion curve tends to bend towards higher incidence angle and higher wavelengths. We also note that a negative slope occurs in the wavelength range above the black dashed lines. Indeed, this specific wavelength range in which negative slope is obtained can be tuned from visible region to IR region by changing geometrical parameters such as the period or grating thickness.

### Application to sensing

In order to compare the potential performance of both configurations for SPR sensing, we have followed the common knowledge in the field by introducing the Figure of Merit (FOM) defined by FOM = S/w, where w and S denote the FWHM of SPR dip and the sensitivity (i.e. the change in resonance wavelength as a function of a change in the refractive index of the dielectric layer on top of the metal) respectively^39–41^.

**Figure 6:**
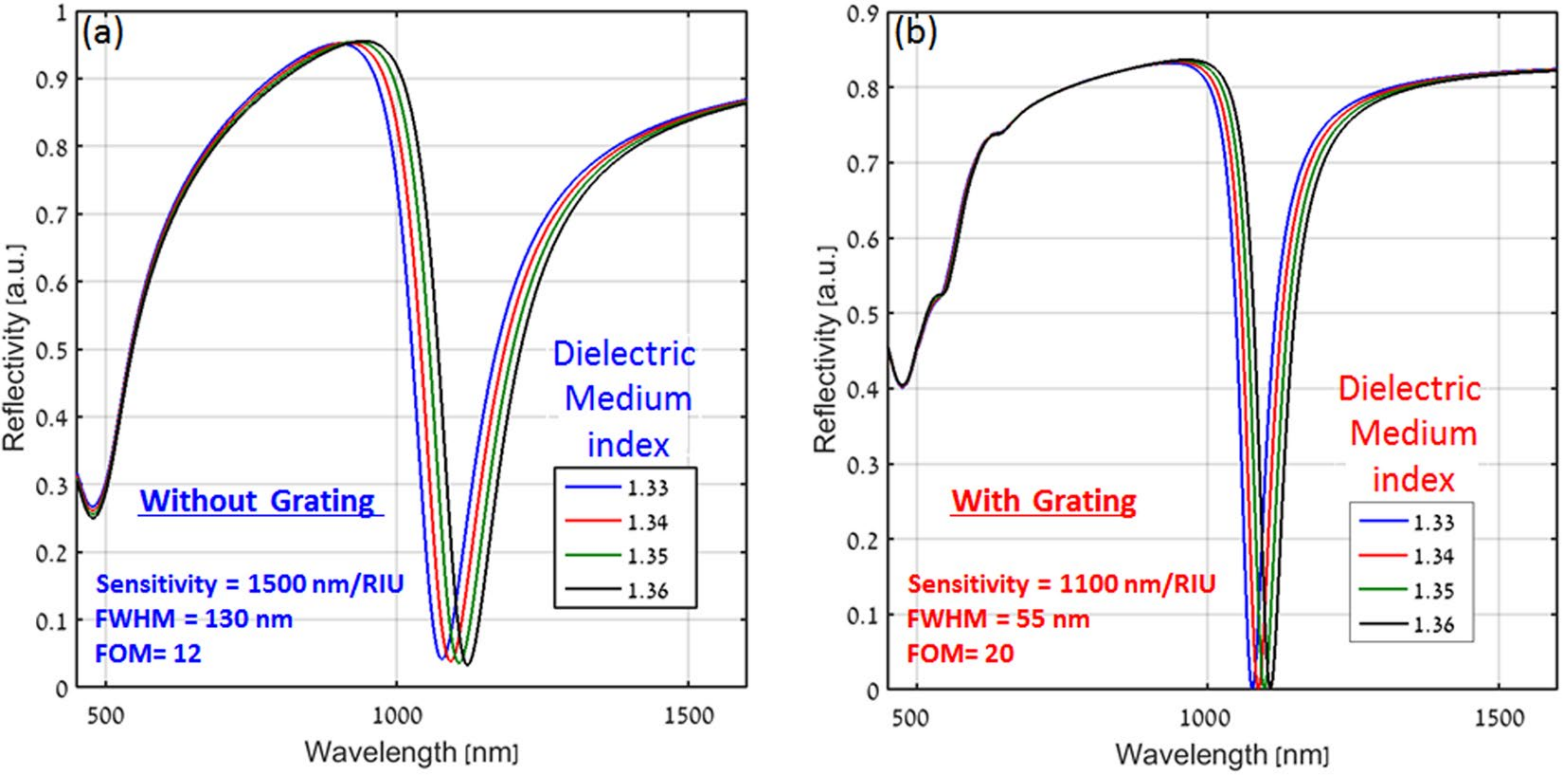
Calculated reflectivity spectra for several choices of top dielectric layer for (**a**) Conventional and (**b**) Plasmonic nanograting based Kretschmann configurations. A dielectric film of thickness 100 nm is considered on the top layer of both the configurations and the reflectivity spectra was calculated with the increase in the refractive index of the dielectric film.

To calculate the sensitivity for both the configurations, a dielectric layer of thickness 100 nm was assumed to be placed on the top surface. The refractive index of this layer was gradually varied from 1.33 to 1.36 in step of 0.01. Figure 6(a,b) show the reflectivity spectra for several choices of refractive index of the dielectric layer for the conventional SPR and the plasmonic nanograting configurations, respectively. For both cases, a red shift in the spectra is observed with the increase in the refractive index of dielectric layer.

As can be seen, the sensitivity (S) for the conventional prism configuration is higher (1500 nm/RIU) than that of the nanograting configuration (1100 nm/RIU). Yet, due to its high Q factor (or equivalently the smaller value of w, 55 nm versus 130 nm), the plasmonic nanograting still provides a better FOM (20 > 12) as compared to the conventional configuration. As such, one may expect better sensing capability, i.e. the plasmonic nanograting configuration is expected to provide the capability of observing smaller changes in the refractive index of the top layer, and as such, it should be sensitive to a smaller concentration of analytes.

**Figure 7:**
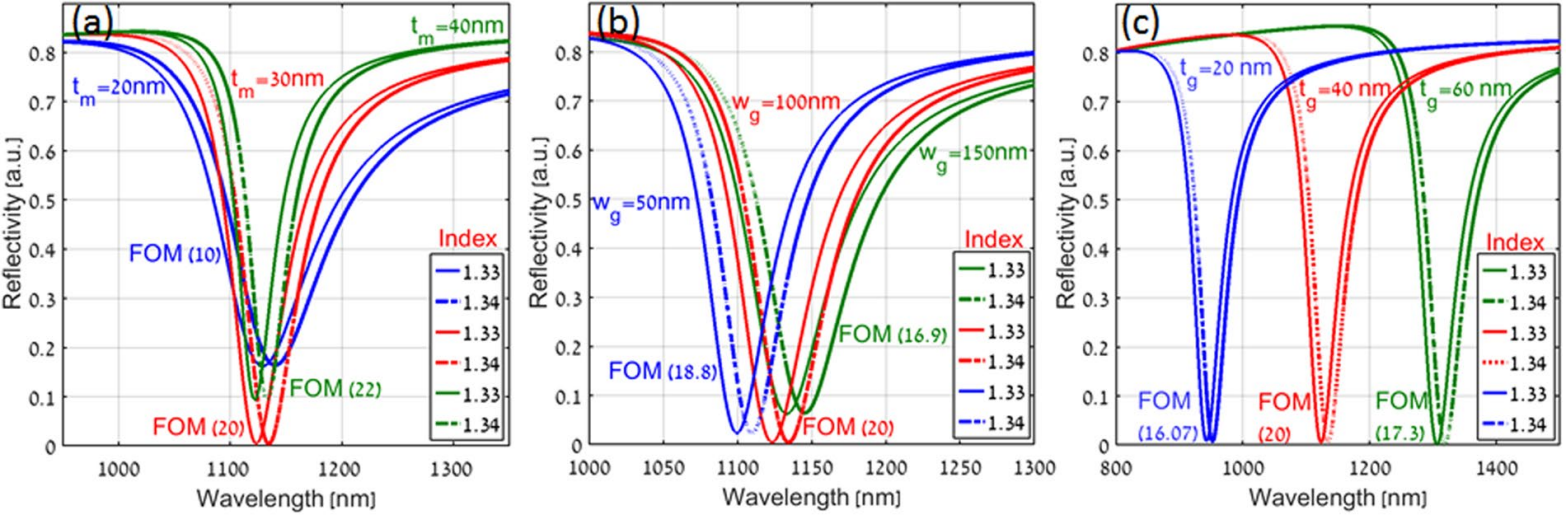
Calculated reflectivity spectra of our plasmonic nanograting based Kretschmann configuration for different values of refractive index as a top dielectric layer with (**a**) variation in thin metal height (t_m_) (**b**) variation in grating width (w_g_) and (**c**) variation in grating height (t_g_) respectively.

To show the effect of different geometrical parameters on the enhanced FOM for plasmonic nanograting based Kretschmann configuration, the reflectivity spectra was calculated for different values of refractive index as a top dielectric layer with variation in the geometrical parameters as shown in Fig. 7. Figure 7(a) shows the reflectivity spectra for two different refractive indices (1.33 and 1.34) of the top dielectric layer with variation in thin metal height (t_m_) of the plasmonic nanograting configuration. As expected, a red shift in the spectra was observed with the increase in the refractive index of the top dielectric layer. For metal height of 20 nm (blue color), the broad linewidth (FWHM) for SP resonance resulted in FOM value of 10, whereas, for 40 nm thick metal (green color), the value of FOM was found to be 22 due to narrower FWHM. The optimized 30 nm metal height (red color) exhibited about the same FOM (20) while offering the highest signal contrast.

Figure 7(b) shows the reflectivity spectra for two different refractive indices (1.33 and 1.34) of the top dielectric layer with variation in grating width (w_g_) of the plasmonic nanograting configuration. It can be noticed that for the optimized 100 nm grating width (red color), the narrowest FWHM with a higher signal contrast resulted in FOM value of 20, which is higher as compared to other two cases (blue and green color) shown in Fig. 7(b).

Figure 7(c) shows the reflectivity spectra for two different refractive indices (1.33 and 1.34) of the top dielectric layer with variation in grating height (t_g_) of plasmonic nanograting configuration. It can be noticed that for values of t_g_ = 20 nm (blue color) and 60 nm (green color) the FOM values found to be 16.07 and 17.3 respectively, whereas, for optimized value of t_g_ = 40 nm (red color), the narrowest FWHM of SP resonance resulted in FOM value of 20.

## Experimental Results

**Figure 8:**
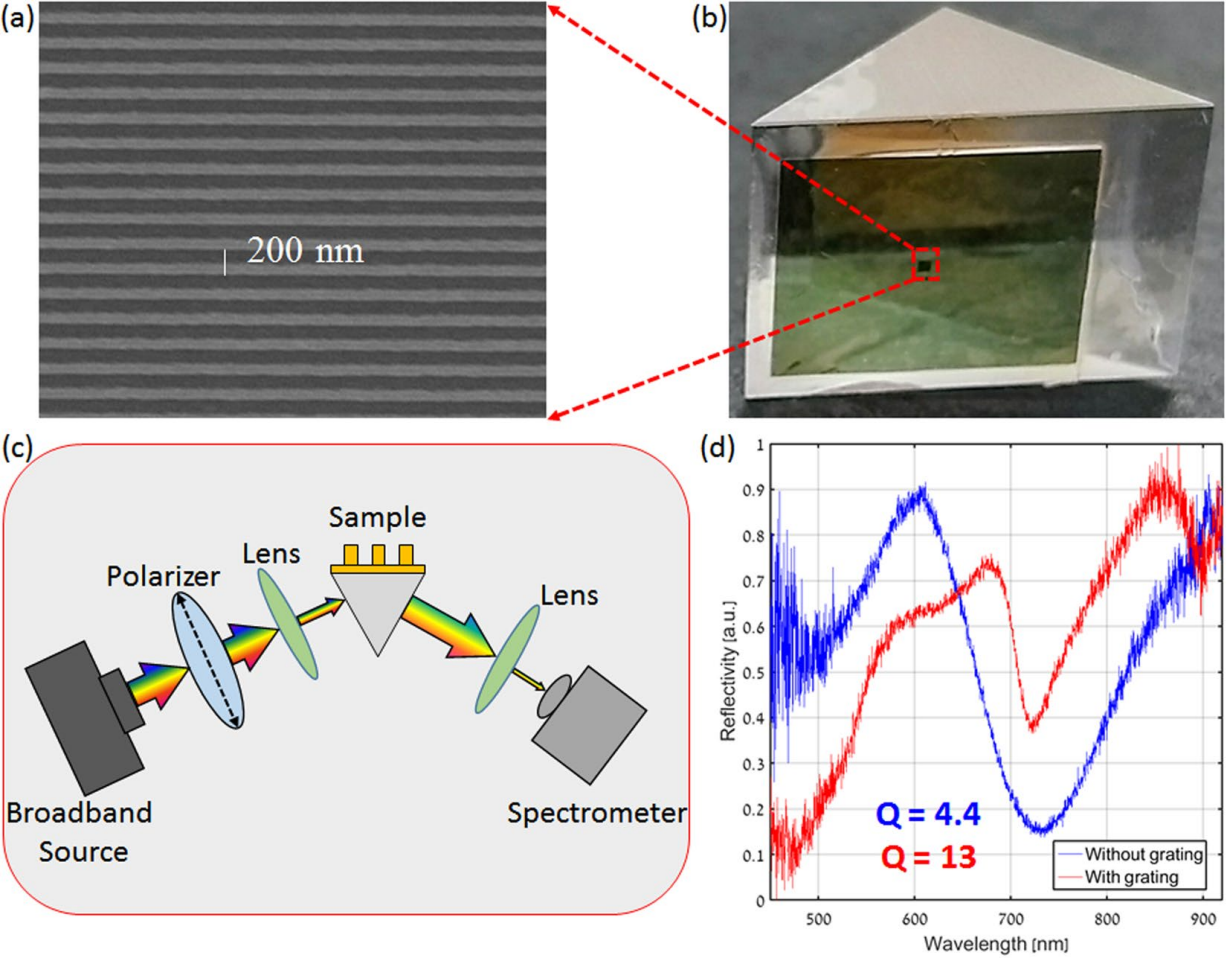
(**a**) SEM Micrograph of the fabricated 1D plasmonic nanograting with period of 200 nm (**b**) a micrograph showing the integrated plasmonic nanograting glued on top of the prism (**c**) Schematic drawing of the experimental SPR setup. A TM polarized beam from the broadband source is incident on the sample and the reflectivity is measured by the fiber-coupled spectrometer (**d**) Experimentally measured reflection spectra of the samples without (blue) and with (red) the plasmonic grating at incident angles of ~46.2° and ~83°, respectively.

The SEM micrograph for 1D plasmonic structure with period 200 nm is shown in Fig. 8(a). Figure 8(b) shows the photograph for the prism integrated with the plasmonic nanograting. Following the fabrication of the device, we turn into their experimental characterization. Figure 8(c) shows the schematic for the experimental setup for the Kretschmann configuration. A broadband light source in the wavelength range spanning from 400 nm to 1500 nm was used to excite the SPP wave on the sample which is mounted on a rotational stage. TM polarized light focused using a low NA lens was incident on the sample at a fixed incident angle. The reflected light is collected by another lens, and focused towards a fiber-coupled spectrometer (FLAME-S-VIS-NIR-ES from Ocean Optics).

Figure 8(d) shows the experimentally measured reflectivity spectra for both configurations. From the spectra, the reflectivity dip for the sample with the plasmonic nanograting (red line) was found to be significantly narrower than that obtained for the conventional prism configuration (blue line). In terms of Q factor, the plasmonic nanograting configuration (Q = 13) was found to be almost three times compared to the conventional configuration (Q = 4.4) which is in good agreement with the results predicted from the numerical results.

Next we turn into experimentally studying the effect of varying the incident angle on the response of the device. To do so, light from the same broadband source was incident at different incident angles, and the reflectivity spectra were collected for each incident angle.

**Figure 9:**
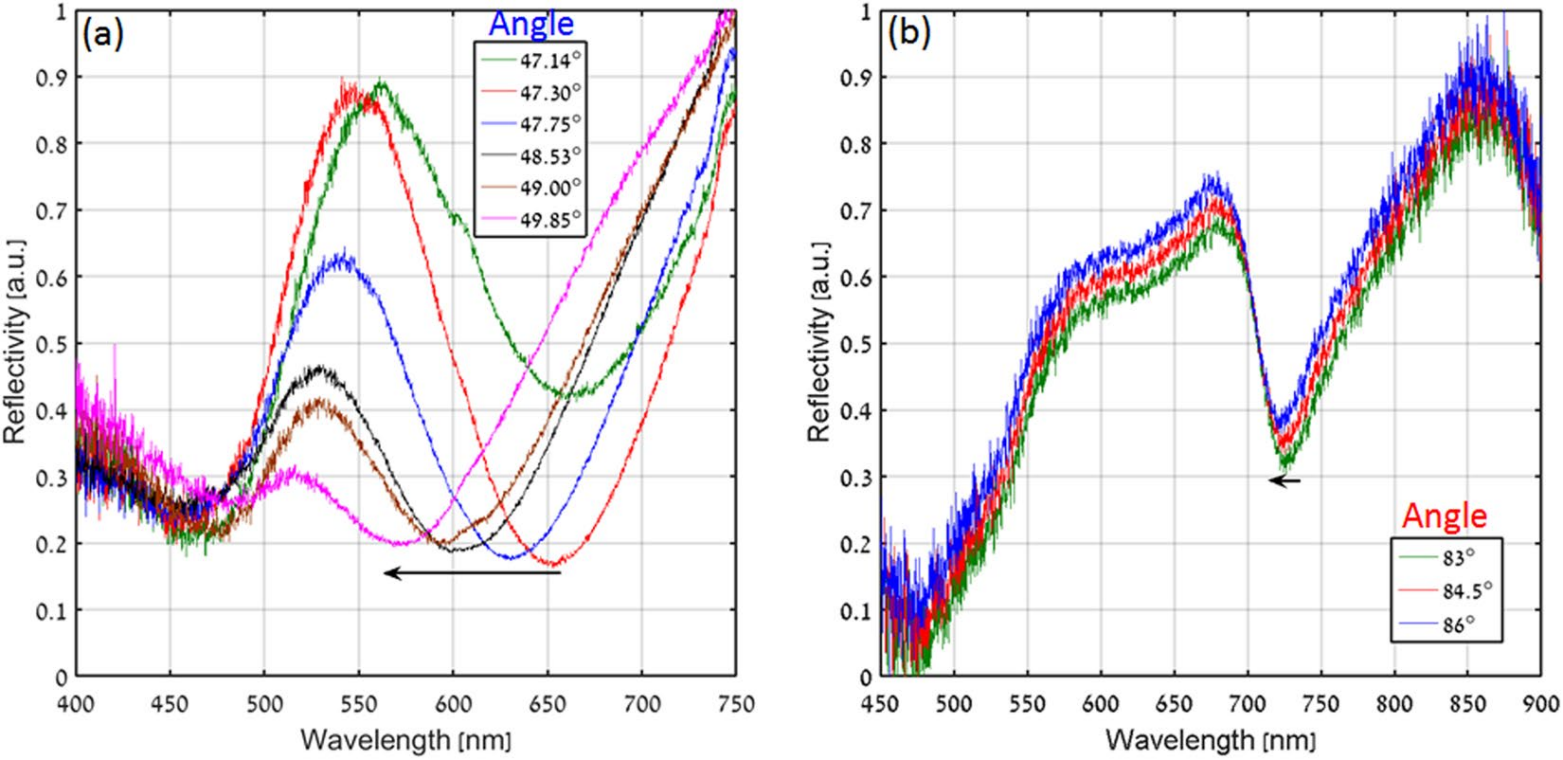
Measured reflected spectra for (**a**) Conventional and (**b**) Plasmonic nanograting based Kretschmann configuration with variation in angle of incidence.

Figure 9(a,b) show the experimentally measured reflectivity spectra for various angles of incidence in both the Kretschmann and the plasmonic nanograting configurations, respectively. As expected from the theory and numerical results, we found the resonance wavelength to decrease with the increase in incident angle for the case of the Kretschmann configuration (Fig. 9(a)), whereas, one observes a negligible wavelength shift for different incident angles for the plasmonic nanograting configuration. Indeed, this is the direct result of our dispersion engineering approach, implemented by the plasmonic nanograting. By increasing the dispersion, it is possible to minimize the angular sensitivity of the resonance wavelength which can be helpful for studying and utilizing light-matter interaction effects with a narrow linewidth media (e.g. Rb atom or molecule) having nonlocalities in their susceptibility-momentum relation^42^. For such cases, the proposed plasmonic nanograting device can be used as a robust system to keep the resonance wavelength constant for broad angular (momentum) range without shifting away of the resonance frequency of the media.

## Conclusions

We have shown numerically and experimentally that the resonance linewidth in an SPR based on the Kretschmann configuration can be reduced by using the concept of dispersion engineering. More specifically, by incorporating a plasmonic nanograting on top of a flat metal film we obtained a slow light effect, leading to a three to four-fold increase in Q factor. The plasmonic nanograting allows to tailor the dispersion of the plasmonic mode such that dispersion engineering and high group index is achieved. We have also shown that the figure of merit for sensing can be improved, essentially allowing to detect smaller changes in refractive index and hence to observe lower concentration of analytes. Furthermore, we have calculated the electromagnetic field distribution and show that the plasmonic nanograting further confine the field, offering the advantage of enhanced light matter interactions. Another important aspect of our device is the reduced sensitivity to angle of incidence. While in the conventional Kretschmann configuration the resonance wavelength shifts significantly with the angle of incidence, this is not the case for our configuration due to the high group index. As such, our device may become useful in probing atoms or molecules near their resonance wavelength under broader momentum excitation. Although the fabrication using electron beam lithography is considered as limiting factor, this obstacle can be removed by using approaches such as nano-imprint lithography. Moreover, the aspect ratio required for the implementation of our plasmonic nanograting is fairly low (~0.4) which is advantageous for biomedical applications for two main reaons: 1 – it is easier to fabricate and 2 – it still allows to maintain laminar flow of the analyte between the grooves of the structure during the adsorption of the bio-analyte (e.g. antibody or antigen). In the future, we also intend to integrate our device with analytes delivery system such as microfluidic channels, and to probe actual analytes in an absorption spectroscopy scheme. By doing so, it should be possible to achieve an advanced, versatile, robust and low cost integrated sensor for variety of sensing applications.

## Methods

### Fabrication

For comparison purposes, we have fabricated two types of samples, the conventional Kretschmann device and the plasmonic nanograting device. For the Kretschmann configuration, we have used a right-angle CaF_2_ prism. After cleaning with soap water and DI water, a stack of 2-nm thick Cr (as an adhesion layer) and a 35-nm thick layer of gold were evaporated on top of the prism using e-beam evaporator at deposition rates of 0.3 A°/s and 0.5 A°/s respectively. For the second configuration, plasmonic naonograting was fabricated on top of a 180 μm thick CaF_2_ glass substrate, deposited with 2 nm thick Cr followed by 30 nm thick gold layer using e-beam evaporator. Next, a 200 nm thick PolyMethylMethAcrylate (PMMA) 950 K e-beam resist from Microchem was spin coated over the gold-coated sample and patterned using Raith 150^TWO^ electron beam system with 20 kV accelerated voltage, 10 mm working distance, 10 μm aperture size and an optimized dose of 500 μC/cm^2^ for patterned area 600 μm × 600 μm. The exposed PMMA was developed for 60 seconds using MIBK:IPA (1:3) solution in cold environment. A 40 nm thick gold layer was deposited on the patterned PMMA grating using e-beam evaporator. Lift off was used to obtain designed structure by treating the sample with acetone using ultrasonic-bath for 5 minutes. The plasmonic nanograting fabricated on glass substrate was integrated on the prism with index matched (NOA-85) optical adhesive.

### Data availability statement

The dataset generated or analyzed during the current study are available from the corresponding author on reasonable request.
